# Genome Analysis of *Staphylococcus aureus* ST291, a Double Locus Variant of ST398, Reveals a Distinct Genetic Lineage

**DOI:** 10.1371/journal.pone.0063008

**Published:** 2013-05-21

**Authors:** Marc Stegger, Maliha Aziz, Tomasz Chroboczek, Lance B. Price, Troels Ronco, Kristoffer Kiil, Robert L. Skov, Frederic Laurent, Paal S. Andersen

**Affiliations:** 1 Microbiology and Infection Control, Statens Serum Institut, Copenhagen, Denmark; 2 Translational Genomics Research Institute, Pathogen Genomics Division, Flagstaff, Arizona, United States of America; 3 Department of Environmental and Occupational Health, George Washington University, Washington, D. C., United States of America; 4 National Reference Center for Staphylococci, Laboratory of Bacteriology, Hôpital de la Croix Rousse, Inserm U851, Lyon, France; University of Edinburgh, United Kingdom

## Abstract

*Staphylococcus aureus* ST291 has been reported as a homologue recombinant double locus variant of the livestock associated *S. aureus* ST398. However, whole genome sequencing show that ST291 is a unique genetic lineage with highly variable content within its accessory genome compared to both human and livestock associated genome sequenced CC398s.

## Introduction

Since its discovery, there has been a great interest in *Staphylococcus aureus* isolates related to clonal complex (CC) 398, both in terms of epidemiology and diversity of its genomic content [1]–[5]. To facilitate rapid detection of *S. aureus* CC398 isolates, we recently developed a PCR targeting the CC398-specific variant of *sau1-hsdS1*, a gene encoding the specificity subunit of the *S. aureus* restriction-modification system Sau1 [6]. *S. aureus* isolates of sequence type (ST) 291, have been assumed to be double locus variants (DLV) of CC398, potentially as a result of a homologue recombination event as indicated by multi locus sequence typing (MLST) [7]. ST291 has been reported to be sensitive to *Sma*I digest and thus different from the normal CC398 population by standard pulsed-field gel electrophoresis (PFGE) procedures, and also to exhibit major differences in *spa* repeats compared to other published CC398-associated *spa* types. It has also escaped detection by ourCC398 specific PCR [6]. In a recent study using whole genome sequence data to analyze the population structure of CC398, no ST291s were included to give insight into this specific subgroup of CC398 [5]. ST291 isolates have been scarcely described in the literature from areas associated with the presence of CC398, such as France, Italy, Switzerland, and the US, but recently more abundant in areas that have not previously been associated with CC398 such as England, India, Iran, Korea, Lebanon, Mali, and Tunisia [7]–[17], including reports of invasive, methicillin-susceptible as well as methicillin resistant, variants. To further understand and characterize how this reportedly DLV of ST398 differ from the general population structure of CC398 we performed whole genome sequencing (WGS) of two ST291 isolates.

## Materials and Methods

### MLST analysis

A population snapshot using eBURST at the MLST database (http://eburst.mlst.net/) identified STs either directly linked to CC398 or being DLV of sequence types linked to CC398 (data not shown). A maximum parsimony analysis using MEGA 5.05 [18] of the concatenated MLST alleles from the most closely related available *S. aureus* genome to ST398, MRSA252 (GenBank accession no. NC_002952), and the identified STs was used to evaluate the MLST allele based population structure of *S. aureus* CC398.

### DNA sequencing and typing

Two invasive MSSA representatives of the ST291 genotype, HT20040853 and ST20090964, from France (*spa* type t3642) and Algeria (*spa* type t779), were sequenced by paired-end Illumina sequencing with 101-bp read lengths as previously described [5]. The two isolates were *in silico* MLST typed using assembled whole genome sequence data at http://cge.cbs.dtu.dk/services/MLST [19]. The Illumina whole genome sequence data for the two ST291 isolates are available at the Short Read Archive with accession ID SRA060904, with ∼200 fold coverage. The accession numbers for the previously sequenced Illumina sequence data generated from 88 *S. aureus* CC398 isolates are available in the Sequence Read Archive under the following accession numbers: SRX129593 to SRX129632, SRX129682 to SRX129686, SRX129691, SRX129696, SRX129697, SRX129701, SRX129702, SRX129704 to SRX129707, SRX129714, SRX129718, SRX129758, SRX129763, SRX129764, SRX129766, SRX129775, SRX129779, SRX129784, and SRX129816 to SRX129840.

### *De novo* and reference assembly

Reference assembly against the MRSA livestock associated S0385 ST398 genome and the MSSA human associated ST398NM01/71193 ST398 genome (GenBank accession no. AM990992 and CP003045, respectively) was performed using CLCbio's Genomics Workbench 5.5.1 (CLCbio, Aarhus, Denmark). *De novo* assemblies of both the unmapped reads from the reference assemblies as well as on the entire data sets were also performed using the Genomics Workbench.

### SNP calling

Single nucleotide polymorphisms (SNPs) was identified as described previously [5]. Briefly, in order to avoid false calls due to sequencing errors, SNP loci were excluded if they did not meet a minimum coverage of 10× and if the variant was present in less than 90% of the base calls for that position. SNP calls were combined for all of the sequenced genomes such that for the locus to be included in the final SNP matrix, it had to be present in all of the genomes. SNPs in the duplicated regions on the reference genome were discarded.

### Genome analysis

Comparative genomics was performed using reference assemblies against the MRSA livestock associated S0385 ST398 genome and the MSSA human associated ST398NM01/71193 ST398 genome and gaps were identified in regions where there was no coverage (missing genes/genomic regions) and by subsequent *de novo* assembly of unmapped reads from the reference assemblies were performed using CLCbio's Genomics Workbench 5.5.1 (CLCbio) thereby identifying genes/genomic regions present in ST291 but absent in CC398. The distinct, non-ST398 mobile genetic elements (MGE) were annotated and compared using the annotation pipeline and sequence based comparison tool at RAST (http://rast.nmpdr.org) [20]. For visualization, the ST291 WGS data sets were aligned against the two *S. aureus* ST398 reference genomes (GenBank accession no. AM990992 and NC_017673), using BWA version 0.6.2-r126 and SAMtools version 0.1.18 [21], [22]. BLAST atlases comparing the two reference sequences were generated using GeneWiz with an additional lane added to display the read depth of the aligned reads [23].

## Results and Discussion

**Figure 1:**
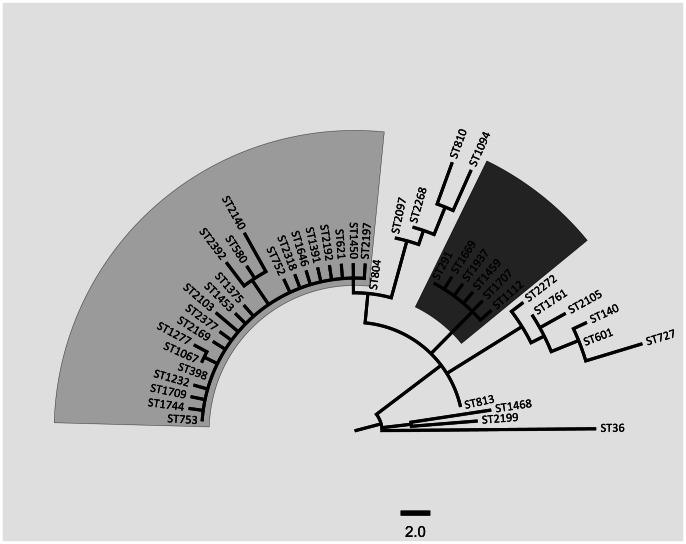
MLST based CC398 phylogeny. Maximum parsimony analysis of the concatenated allelic sequences from the 43 CC398 sequence types and rooted using MRSA252 (ST36). The cluster containing ST398 is highlighted in light grey and the ST291 cluster is dark grey. The scale indicates the number of SNP differences.

**Figure 2:**
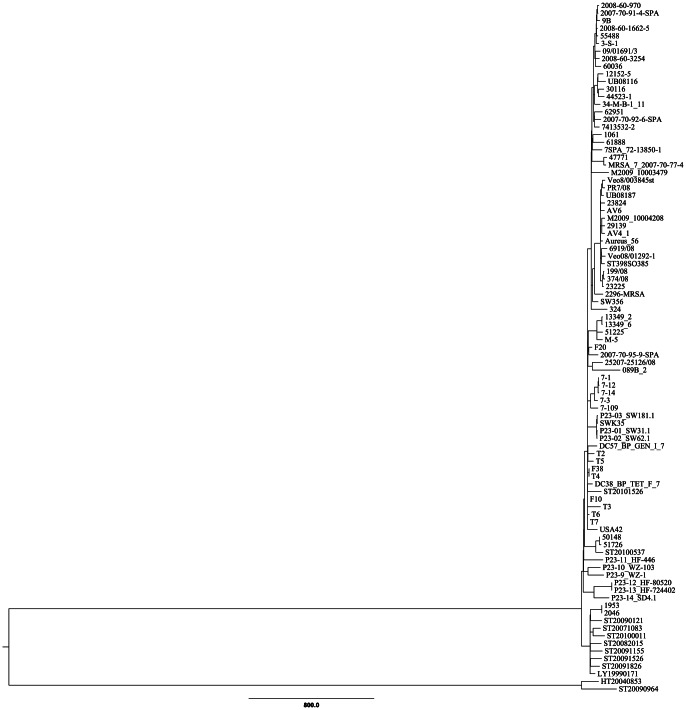
Whole genome sequence based analysis of divergence of ST291 and ST398. Maximum-parsimony tree of the two ST291 isolates (HT20040853 and ST20090964) and 89 CC398 isolates based on 12,735 SNPs total SNPs. The tree was rooted with MRSA252 (ST36), the closest non-CC398 available out-group. The scale indicates the number of SNP differences.

**Figure 3:**
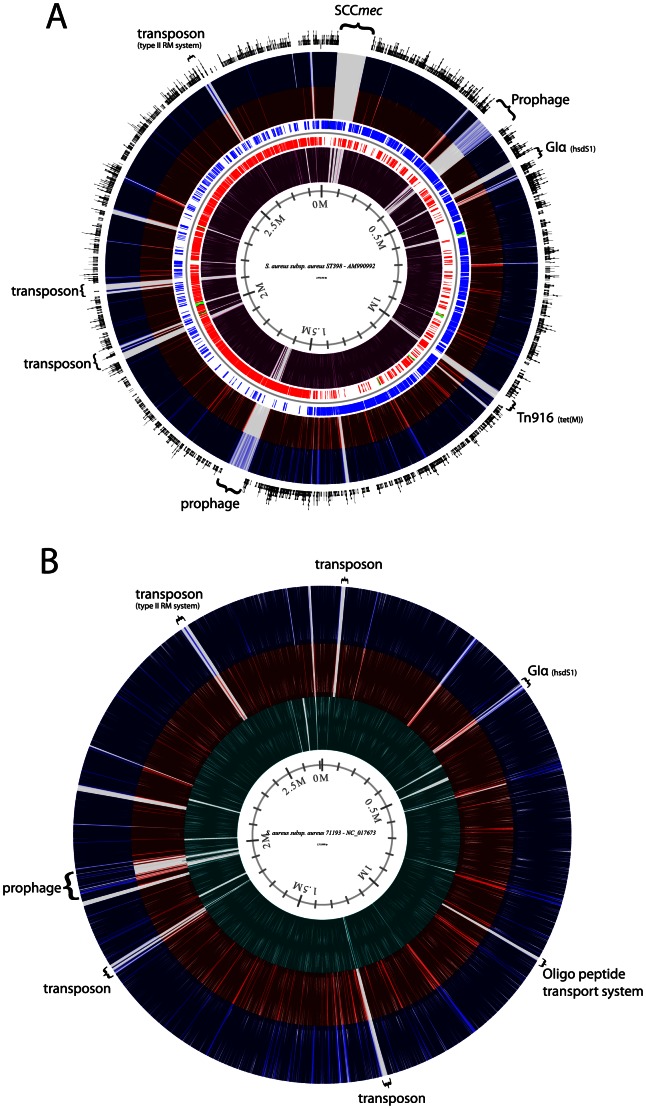
Diversity in *S. aureus* CC398 genome content. Comparison of the genome content of the livestock- and human-associated *S. aureus* CC398s with the two ST291s. Reference genomes are in A) S0385 the livestock associated CC398, and in B) ST398NM01/71193 the human associated CC398. Presented are analysis of ST398NM01/71193 (purple), S0385 (cyan), HT20040853 (blue) and ST20090964 (red), compared to the references. The outer circle in A represent the distribution of the 7,800 SNPs differentiating both ST291s from the SO385 reference genome.

Phylogenetic analyses of the concatenated MLST alleles readily identified several sub-clusters within the *S. aureus* CC398 complex, with the presence of a distinct ST291 clade comprised of six different STs as seen in Figure 1. A total of 12,735 SNPs were identified among the entire 91 CC398 isolates, and with 11,744 identified SNPs after exclusion of the 123 kb horizontally acquired ST9 region. The maximum parsimony phylogenetic tree presented in Figure 2 based on the 11,744 SNPs revealed that ST291 formed a very distal cluster in relation to the previously published 89 CC398 genomes, with no evidence of any major horizontal gene transfers (HGTs) to account for the observed SNP diversity (see Figure 3). A total of 7,800 shared SNPs separated both ST291s from the S0385 CC398 reference genome, and only 329 SNPs differentiated the two ST291 isolates. In reference, the phylogeny on the 89 previously published *S. aureus* CC398 genomes used to evaluate the overall population structure of *S. aureus* CC398 was based on a total of 4328 SNPs [5].

CC398 encodes a single *sauI-hsdS1* gene located in the genomic island (GI) α, whereas most other *S. aureus* lineages encode two distinct variants of *sauI-hsdS*; *sauI-hsdS1* and *sauI-hsdS2* located in GIα and GIβ, respectively [1], [24]. ST291 also encode two specificity subunits, with the *sauI-hsdS1* showing ∼60% nucleotide similarity to the CC398 *sauI-hsdS1*, and consequently the CC398 specific PCR do not identify these isolates as part of the CC398 cluster. The *sau1-hsdS1* has a very high sequence similarity (>99%) to a *sau1-HsdS* gene present in a non-GI associated restriction-modification system present in the genome sequence of a representative of the European community acquired ST80 lineage [25]. Additionally, ST291 isolates have been reported as being capable of *Sma*I restriction cleavage in pulsed-field gel electrophoresis analysis unlike CC398 [8].

Comparative genomics show that an extensive number of MGEs are absent from the two genome sequenced ST291 isolates compared to both the human and livestock associated ST398 (see Figure 3). These MGEs include both larger regions such as the SCC*mec*, multiple transposon and phage elements, but also “core genome” encoded adhesins, lipoproteins, various enzymes and a number of hypothetical proteins. A significant proportion of the genomic island α is also lacking from the ST291 genomes when compared to the two representative ST398s, including the gene encoding the von Willebrand factor-binding protein, in addition to harboring the divergent *sauI-hsdS1* gene. A large proportion of the MGEs that seems to have been acquired at least in the representative livestock associated CC398 reference genome compared to reference human associated CC398 genome are also absent from our two ST291 genomes, see Figure 3A. We do however also see that multiple MGEs are unique in the human associated reference genome both compared to the livestock associated representative genome and to the ST291 genomes, see Figure 3B. *De novo* assembled contigs from unmapped ST291 reads from reference assemblies against the human associated ST398NM01/71193 ST398 genome sequence identified approximately 140 and 205 kb of novel content on 39 and 54 contigs >500 bp in ST20090964 and HT20040853 respectively, see Supplementary files S1 and S2. These *de novo* contigs encode a high number of phage related proteins, but also membrane transport systems, an ESAT-6 gene cluster, serine proteases, multiple components of restriction modification systems, and replication related proteins indicating at least one plasmid in each of the two isolates. The genomes of the two ST291 isolates contain the staphylococcal epidermal cell differentiation inhibitor (EDIN) exotoxin, which has been associated with promoting formation of infection foci in deep-seated tissues [26]; and both isolates encode SCIN, also suggesting them to belong to a human associated clonal lineage [5], [27]. Additionally, the French ST291 isolate carries the *lukS*/*F*-*PV* operon encoding the Panton-Valentine Leukocidin.

ST291 is the fifth most abundant carrier clone in Mali [17], and searching the Ridom Spa Server (http://spa.ridom.de) for *spa* types related to t3642 (e.g. t937, t1149, t1614, t2313, t2993) show the widespread presence of both methicillin-susceptible and methicillin-resistant strains within the USA, Europe, and the Middle East. Identifying *spa* types related to t779 with its single repeat (r08), or linking them to both a group of STs or even specific clonal lineages may be associated with great error, but *spa* type t779 is reported via the Spa Server from throughout Central and Northern Europe.

## Conclusions

Based on our analysis, ST291 isolates and likely isolates with the STs highlighted in dark grey in Figure 1, are not closely related to ST398 or to the livestock associated CC398 group in general. Concordantly, we suggest when reporting CC398s related to livestock, that isolates related to ST291 should not be grouped as part of this cluster. The reported ST291 furthermore shows a noticeably different geographic dispersion compared to ST398, indicating a distinct epidemiology possible linked to a community associated human lineage.

## Supporting Information

Supplementary File S1 S_aureus_ST20090964_accessory_genome.gbk.
(GBK)

Supplementary File S2 S_aureus_HT20040853_accessory genome.gbk.
(GBK)
